# 

**Published:** 1868-10

**Authors:** 


### CONTENTS.

ORIGINAL COMMUNICATIONS.

The Life of the Trichina........ 399
Alveolar Abscess........ 412
Le Miserable........ 421

PROCEEDINGS OF SOCIETIES.

Extracts from Proceedings of the Indiana State Dental Association........ 425

EDITORIAL.

Plastic Gold........ 436

Treatment of Roots........ 438

Instruments........ 440

Instinct Outstunk........ 441

Private Correspondence........ 442

